# Systemic problems require systemic solutions: the need for coordination and cooperation to improve research quality

**DOI:** 10.1186/s13104-022-05932-5

**Published:** 2022-02-14

**Authors:** Emma Ganley, Anne-Marie Coriat, Sarah Shenow, David Prosser

**Affiliations:** 1 protocols.io, Perth, UK; 2 Wellcome Trust, London, UK; 3 Mental Health Research, London, UK; 4 Research Libraries, London, UK

**Keywords:** Reproducibility, Replicability, Research quality, UKRN

## Abstract

Various factors contribute to low reproducibility and replicability of scientific findings. Whilst not all of these are necessarily problematic, there is growing acceptance that there is room for improvement. Many sectoral organisations have a role to play in this, by refining incentives and rewards, promoting specific behaviours such as open research practices, and exploring innovations in grant funding and scientific publishing. However, given the systems nature of the challenge, real change will require the coordination of these efforts, and partnerships that ensure alignment of activity and interoperability of training. Efforts to improve research quality will require investment, in infrastructure, training, and research on research to ensure that innovative solutions are evidence-based, and potential unintended consequences are explored (and avoided). National structures (e.g., the planned UK Committee on Research Integrity) should focus on understanding the research system, identifying areas for improvement, and promoting research to understand the impact of novel approaches and innovations, in order to advise on how to maximise benefit and avoid harm.

## Introduction

The UK Reproducibility Network (UKRN; www.ukrn.org) is a national peer-led consortium that aims to ensure the UK retains its place as a centre for world-leading research. UKRN includes external stakeholder, comprising funders, publishers, learned societies and other sectoral organisations. Currently, 37 organisations are part of the UKRN Stakeholder Engagement Group (https://www.ukrn.org/stakeholders/)—Table 1. This commentary has been developed with the input of the individuals that represent these organisations on the Stakeholder Engagement Group, including drawing on the individual submissions prepared by these organisations, and represents the position of this part of the UKRN structure.

**Table 1:** The UKRN Stakeholder Engagement Group (as of Jan 14th 2022)

THE UKRN Stakeholder Engagement Group	
Full stakeholders, who provide direct funding for UKRN activities, as well as support in kind	Academy of Medical Sciences The Arts & Humanities Research Council Cancer Research UK The Economic and Social Research Council Jisc The Medical Research Council MQ: transforming mental health The Natural Environment Research Council Research England United Kingdom Research and Innovation UK Research Integrity Office The Wellcome Trust
Affiliate Stakeholders, who we work with collaboratively and who provide support in kind for UKRN activities	The Association of Research Managers & Administrators The British Neuroscience Association The British Psychological Society The CHDI Foundation Code Ocean CLOSER The European Bioinformatics Institute EBSCO F1000 Research FAIRsharing The Institute of Physics The NC3Rs The National Institute for Biological Standards and Control The National Physical Laboratory Nature Publishing Group Public Library of Science Project TIER protocols.io Research Libraries UK The Royal Society of Biology The Software Sustainability Institute The UK Data Service The UK Research Staff Association Universities UK Wiley

## Main text

We recognise that low reproducibility and replicability of scientific findings can be caused by many factors, not all problematic. However, there is converging evidence that the robustness of scientific findings, and research ecosystem in general, could be improved. Reflecting on how we can do this is positive, and since 2019 UKRN has attempted to coordinate efforts to do this. It is also important to recognise that some potential solutions will apply broadly, whilst others will be more discipline specific. These also will need to be developed with the community of researchers and others within the research ecosystem. If we can improve the quality of the research we produce, and ensure it is adequately recorded, we can reduce waste, and maximise the value of research investment—whether in terms of advancing of knowledge or having a positive impact on society.

In our view, several factors can contribute to non-reproducibility or non-replicability, including previously unknown effects that influence the main result, inadequate recordkeeping, technology limitations, biases, lack of training, institutional barriers, or even misconduct, in rare cases. However, the recent National Academy of Sciences report on *Reproducibility and Replicability in Science* [1] notes that “*Reproducibility is strongly associated with transparency; a study’s data and code have to be available in order for others to reproduce and confirm results.”* Here we present our suggestions for what different stakeholders within the research ecosystem—funders, publishers, learned societies, and other sectoral organisations—could do to help address issues with reproducibility.

## Funders

Funders have an important role to play in improving the quality of research, given the incentives created by the design and delivery of funding schemes, and the impact this has on the behaviour of scientists. For example, requiring sharing of data, code, methodologies and other materials as appropriate, and then monitoring and enforcing this, would strongly encourage open research and research transparency. Funders should also ensure training in research integrity, open research practices, and methodology is supported (for example in doctoral training programmes and fellowships), and could consider—over time-making such training a requirement for the award of funding at all career stages.

Some funders now require explicit reference to ways in which a project will ensure the reproducibility of the results generated (see the UK Medical Research Council’s Reproducibility and Statistical Design annex [2] for example). This could be implemented by other funders, with applicants required to specify this in their applications. It is worth noting that this will require the relevant infrastructure to be available (e.g., digital repositories for data and methods, and electronic notebooks), the training in place to ensure digital deposits of intermediate research artefacts are of a high standard, and monitoring to ensure that what is described in funding applications is delivered. This will require investment.

There is also scope for innovation and coordination with other stakeholders. For example, Registered Reports Funding Partnerships allow for the integration of the funder review process with the journal peer review process, and encourages the uptake of the Registered Reports publishing model (where publication is decided on the basis of the importance of the research question and the robustness of the methodology, rather than on the noteworthiness of otherwise of the findings) [3, 4].

However, it will also be important to conduct research into whether these innovations improve research quality as intended, and whether or not they have any unintended consequences. Funding should be made available to investigate how the research ecosystem works—to build research and development capacity focused on improving the research ecosystem, and understanding how its incentives and rewards impact on research behaviour. By extension, funders should be explicitly supportive of grant funds being used to support research improvement of all kinds, and should consider specific funding mechanisms for this activity. This investment is likely to repay itself by improving the quality of the research we produce, and in turn improving the speed with which this research advances knowledge or benefits society.

## Publishers

Publishers can contribute to the improvement of research quality by ensuring that reviewers have relevant expertise and training, supporting examples of effective practice and guidance, engaging with stakeholders (e.g., researchers, funders) earlier in the research process, moving beyond traditional article formats, supporting open research practices, experimenting with innovative approaches, providing training, and aligning incentives and rewards. Registered Reports Funding Partnerships (above) are an example of an initiative that includes several of these elements.

In terms of incentives and rewards, publishers could **place less emphasis on novelty**. The overwhelmingly positive stories in research publications can incentivise researchers to play down negative results. We need to encourage a more realistic view of what constitutes valuable research. Some journals have introduced policies that protect authors against “scooping”, by offering a period of protection where manuscripts will be considered even if similar findings have recently been published elsewhere [5, 6]. This reduces incentives to be the first to publish, which can result in a hyper-competitive atmosphere that reduces quality.

## Learned societies

Learned societies can contribute to improving research quality in several ways, given that they can play the role of funder and publisher, and have a membership they can engage with to deliver training or embed incentives to promote specific behaviours. Therefore, many of the ways in which funders and publishers can contribute to improving research quality will also apply to learned societies. They also can implement related initiatives (e.g., promoting open research practices) via other activities such as at scientific meetings. For example, the British Neuroscience Association offers pre-registration posters at its annual meeting, and poster “badges” to recognise open research practices [7].

Some learned societies have taken a proactive stance to issues of research quality. For example, the British Neuroscience Association has established a Credibility Advisory Board (https://www.bnacredibility.org.uk/credibility-advisory-board) to provide expertise and guide the activity of the society. Similarly, the Royal Society of Biology has included defined criteria for doctoral training accreditation that include ‘a high level of professional skills in the field of biology, including thoroughness and reliability’. This highlights the role that learned societies can play in undergraduate and postgraduate courses that they accredit, where that have the ability to require training in relevant skills such as open research practices.

The focus on training embeds scientific rigour at an early stage, even at the undergraduate level. For example, the British Psychological Society has supported Dr Katherine Button’s (University of Bath) project to help third-year psychology students collaborate on a replication study for their final year dissertation project. This model includes implementation of open research practices, such as pre-registering the study’s methods and proposed analyses. The Royal Society of Biology has incorporated skills relevant to reproducibility into their accreditation programme, and has also recently strengthened them significantly in revised criteria—under the area of quality management and regulator compliance.

## Other sectoral organisations

A number of sectoral organisations interact with research organisations outside of academia (e.g., the pharmaceutical industry). It is worth noting that research in academia does not have the same drivers as industrial research and development processes to undertake measurements in support of regulatory compliance; this is fundamental to small and medium-sized enterprises (SMEs), for example. However, there is potential to learn from these other industries—for example, the pharmaceutical industry has a robust quality assurance framework intended to ensure data integrity and the quality of results generated in this sector.

Data management compliance is critical to confidence in research outputs, and there is a case to say that research funders should require data management processes to be evident. This would have some significant effects on training, especially at undergraduate and first graduate levels in universities. Learning effective practice across organisations and sectors could drive improvement in research conducted in academia; bringing everyone together should enhance the whole relationship between academic research and the work done in private companies.

There are also a **large number of community initiatives** that have emerged in recent years. For example, FAIRsharing (https://fairsharing.org/) is an educational resource that describes and interlinks community-driven standards, databases, repositories and data policies. Peer Community in is an organization that aims to create communities of researchers recommending preprints in their field (https://peercommunityin.org/). This exists now for many reseach communities including one for registered reports, which is supported by UKRN. There are also a number of initiatives that have been developed by early career researchers, such as the ReproducibiliTea (https://reproducibilitea.org/) journal club format, and the Reproducible Interpretable Open Transparent (RIOT) Science Club (http://riotscience.co.uk/) seminar series. These speak to the grassroots enthusiasm for developing and implementing novel approaches to training and community building. However, to be effective, there will need to be support for these initiatives to be extended (and if necessary adapted) across scientific disciplines, and there will be a need for research into the impact of these initiatives on research behaviour and the subsequent quality of research outputs.

## Outlook

We know there are issues surrounding a lack of reproducibility in some research fields. This is symptomatic of a larger set of issues about the culture of research and a lack of emphasis/reward on the quality of research conducted. There is a great deal we could learn from other industries that rely on public trust and have developed proactive ‘safety cultures’. Academic research is still overly reactive, placing blame on individuals when things go wrong rather than understanding failures of the system. National structures (e.g., the planned UK Committee on Research Integrity) should therefore focus on understanding this system, identifying areas for improvement (e.g., greater adoption of open research practices), and promoting research to understand the impact of novel approaches and innovations. Sustainable systemic solutions will require novel partnerships across sectors and disciplines including the development of infrastructure that is managed professionally and continually improved.

A greater attention to quality and reproducibility at the academic discovery science phase would improve the effectiveness and efficiency of science and the trust in its outcomes, allowing for faster progress in science and in society [8]. However, this will require investment in infrastructure—including both physical and digital infrastructure (e.g., repositories to support the deposition of digital research artefacts) and training to ensure common standards across the sector and high levels of interoperability between disciplines and institutions. Universities have a responsibility to ensure good practice, but can work collaboratively to achieve this more efficiently, effectively and cost-effectively, and will need to be supported by all sectoral organisations that form the research ecosystem. This will require a degree of coordination and cooperation.

## Data Availability

Not applicable.

## Abbreviations

UKRN: The UK Reproducibility Network
SME: Small and Medium Sized Enterprise
RIOT: Reproducible Interpretable Open Transparent
